# Cocaine as an Anæsthetic and Analgetic for Pharynx and Larynx

**Published:** 1885-03

**Authors:** J. E. Boylan

**Affiliations:** Vienna


### Cocaine as an Anæsthetic and Analgetic for Pharynx and Larynx.

VIENNA, November 12, 1884.

The new anæsthetic for the larynx and pharynx, cocaine, introduced by Dr. Edmund Jelinck, in his paper read before the Imperial Society of Physicians, October 24, is being used daily with the most gratifying results in the Vienna throat clinics. Patients who visit the clinics for the first time and those who refuse to tolerate the probe after repeated introductions are chosen to demonstrate its efficacy. Within one to fifteen minutes after the application of the twenty per cent. solution to the larynx and soft palate, endo-laryngeal manipulations are deliberately and safely undertaken, and while the heretofore most available local anæsthetic has only been resorted to after repeated introductions of the probe have failed to allay the objectionable reflex irritability or when early interference was demanded, cocaine is used at once apparently with impunity. In a recent case, after the general anæsthetic (chloroform) had been administered to a resisting child, in the sitting position, it was found impossible to proceed with the operation owing to the accumulation of mucus in the larynx; when the patiant had rallied cocaine was resorted to and the papilloma removed with but little difficulty from the vocal cord.

The ten per cent. aqueous alcoholic solution of the muriate of cocaine as used by Dr. Jelinck is sufficient to allay slight reflex and moderate pain, but for more irritable cases and for operation a twenty per cent. solution must be used.—*J. E. Boylan, M. D., in Lancet and Clinic.*

---

OXYPHOSPHATE OF ZINC.—Dr. Taygart is said to prepare his oxyphosphate of zinc after the following method: Glace—phosphorous acid is dissolved in a considerable excess of water, and by careful application of heat evaporated to a syrup consistency. For powder, pure zinc oxide is brought to a white heat and so maintained for two hours.

